# The effect of combined calcium and cholecalciferol supplementation on bone mineral density in elderly women with moderate chronic kidney disease 

**DOI:** 10.5414/CN107180

**Published:** 2011-12-21

**Authors:** Cortney Bosworth, Ian H. de Boer, Giovanni Targher, Jessica Kendrick, Gerard Smits, Michel Chonchol

**Affiliations:** 1 University of Washington, Division of Nephrology and Kidney Research Institute, Seattle, WA, USA; 2 University of Verona, Department of Medicine, Section of Endocrinology and Metabolism, Verona, Italy and; 3 University of Colorado Denver, Division of Renal Diseases and Hypertension, Aurora, CO, USA

**Keywords:** cholecalciferol, vitamin D3, bone mineral density, chronic kidney disease

## Abstract

Aims: To examine the effect of combined calcium and vitamin D_3_ supplementation on bone mineral density (BMD) in patients with chronic kidney disease (CKD). Methods: We performed a post-hoc analysis of the DECALYOS II, a 2-year randomized, double-blind, placebo-controlled study of 610 women randomized to: calcium-vitamin D_3_ fixed combination, calcium plus vitamin D_3_ separate combination, or placebo. Both active treatment groups received the same daily amount of calcium (1,200 mg) and vitamin D_3_ (800 IU). BMD of the distal radius was measured by single X-ray absorptiometry at baseline, 12 and 24 months. Results: At baseline 47.2%, 36.4% and 16.4% of the study population had an eGFR ≥ 60, 45 – 59, and < 45 ml/min/1.73 m^2^, respectively. Both active regimens vs. placebo markedly increased serum 25-hydroxyvitamin D levels from baseline in all eGFR groups (p < 0.0001). Analysis of variance demonstrated an overall treatment effect on distal radius BMD (p = 0.005), with the active treatment groups showing a lower rate of BMD loss when compared to the placebo group. The effects of the intervention on BMD did not differ significantly according to baseline eGFR (interaction p > 0.22 for all time points). Conclusion: Combined calcium and vitamin D_3_ supplementation was effective in reducing rate of BMD loss in women with moderate CKD.

## Introduction 

Recent data from the National Health and Nutrition Examination Survey (NHANES 1999 – 2004) suggested that the prevalence of chronic kidney disease (CKD) in the United States has increased to ~ 13% and earlier stages accounted for most of the individuals with CKD [1]. While efforts to delay progression of kidney disease are crucial, it has also been suggested that the early and aggressive management of abnormalities of bone and mineral metabolism are necessary to reduce the morbidity and mortality of patients with CKD [2, 3]. As kidney function declines, low serum levels of 25-hydroxyvitamin D (25(OH)D) are very common in people with CKD [4, 5]. Concomitantly, a decline in glomerular filtration rate has also been identified as an important risk factor of bone loss and fractures [6]. 

Bone mineral density (BMD) and serum 25(OH)D levels are highly correlated to each other when measured across many segments of the general adult population [7]. In addition, meta-analysis data suggest that adequate supplementation with oral vitamin D_3_ (i.e., cholecalciferol) reduces the incidence of hip fractures across a number of population groups [8]. Furthermore, Elder and colleagues showed a significant positive relationship between serum 25(OH)D levels and BMD in patients with Stage 5 CKD, 85% of whom were on chronic dialysis [9]. However, to our knowledge, the effect of vitamin D_3_ replacement on BMD in people with early stages of CKD has been less defined. 

Given that vitamin D_3_ deficiency contributes to an impaired bone health in patients with CKD [10], who have limited ability to convert 25(OH)D to 1,25(OH)_2_D, we undertook a post-hoc evaluation of the DECALYOS (vitamin D_3_, calcium, Lyon) II study performed in French elderly women [11] with the aim of examining the effect of combined calcium and vitamin D_3_ supplementation (cholecalciferol) on radius BMD in subjects with moderate CKD and severe vitamin D_3_ deficiency. 

## Methods 

### DECALYOS II Database

We obtained a copy of the database of the DECALYOS II by submitting a written request to Merck KGaA (Darmstadt, Germany). None of the authors are members of the DECALYOS investigators and thus none was involved in the conduct or initial analysis of the trial. 

### Study population

The recruitment methods, randomization, follow-up and safety results of the DECALYOS II study have been previously described [11]. Briefly, DECALYOS II was a 2-year, multicenter, double-blinded, placebo-controlled study to evaluate the effect of combined calcium and vitamin D_3_ supplementation on radius BMD and secondary hyperparathyroidism in elderly institutionalized women. Subjects were excluded if they were not ambulatory, were not expected to live at least 2 years, had intestinal malabsorption, hypercalcemia (> 10.5 mg/dl) or a serum creatinine concentration > 1.7 mg/dl. Subjects were also excluded if they had received drugs known to alter bone metabolism within the past year (e.g., corticosteroids, anticonvulsants, and high dose thyroxine) or if they had been treated with fluoride salts (> 3 months), bisphosphonates, calcitonin (> 1 month), calcium (> 500 mg/d) or vitamin D (> 100 U/d) during the previous 12 months [11]. 

Enrollment consisted of 610 women living in apartment houses for the elderly. These participants were randomly assigned either 1 of the 2 active treatment groups (calcium-vitamin D_3_ fixed combination group or separate calcium and vitamin D_3_ supplements group) or to the placebo group. The sachet of the calcium-vitamin D_3_ (Ca-D_3_) fixed combination (Ostram-vitamin D_3_, Merck KGaA) contained a fixed combination of 1,200 mg of elemental calcium in the form of tricalcium phosphate and 800 IU of vitamin D_3_ in a single tablet. In the group that received separate tablets of calcium and vitamin D_3_ (Ca + D_3_), the calcium (Ostram, Merck KGaA) content was 1,200 mg of elemental calcium in the form of tricalcium phosphate and the vitamin D_3_ (Devaron, i.e., cholecalciferol, Durphar Solvay) was given in 2 tablets of 400 IU each [11]. 

At baseline, data on each subject’s relevant medical history, diet, and treatments prescribed in the past year, and mobility was recorded. A general physical exam was also performed. Reassessment of medical status was performed every 3 months [11]. 

### Bone mineral density assessments

BMD was measured at the distal radius by single X-ray absorptiometry (SXA) using a mobile Hologic DTX100 Osteometer operated by two nurses recruited and trained for the study. The SXA scanners were calibrated daily. Cross-calibration of the two scanners was performed monthly, and monthly quality control was performed on the scanners by the central laboratory (Synarc, Lyon, France). Longitudinal variability was expressed as a coefficient of variation (CV) using phantom data and estimated at 0.66% and 0.76%. Short-term in vivo precision was estimated at 1.4% (CV) for repeated baseline distal forearm BMD measurements [11]. 

### Biochemical measurements

Laboratory tests were performed after an overnight fast at each investigation site and sent to a centralized laboratory for measurement [11]. All samples were kept frozen at –70 °C until analysis. Serum levels of 25(OH)D were measured using an INCSTAR 25(OH)D 2 step assay procedure with a CV of less than 10%. The first step in the procedure involves the rapid extraction of 25(OH)D from the serum using acetonitrile. Following extraction, the treated sample is assayed by using an equilibrium radioimmunoassay procedure. This method is based on an antibody with specificity to 25(OH)D. The sample, antibody, and tracer are incubated at 20 – 25 °C for 90 minutes. A second antibody-precipitating complex is used to achieve phase separation. This method does not recognize 25(OH)D_2_ and 25(OH)D_3_ separately. The normal range for adults given by the manufacturer of the kit (Incstar) is 15 – 50 ng/ml. Serum intact parathyroid hormone (iPTH) was measured by immuno-chemoluminometric assay (Ciba-Corning Diagnostic) and bone-specific alkaline phosphatase was measured by a two-site radioimmunoassay (Tanden-R-Ostase kit, Beckman-Coulter, CA, USA). Standard laboratories methods were used for the measurements of serum calcium and phosphate [11]. 

### Kidney function

Level of kidney function was defined by estimated glomerular filtration rate (eGFR) using the formula developed and validated in the Modification of Diet in Renal Disease (MDRD) study [12]. The 4-variable MDRD study equation is as follows: eGFR = 186.3 × (serum creatinine^–1.154^) × (age^–0.203^) × 1.212 (if black) × 0.742 (if female). Serum creatinine was assessed by the Jaffé rate-blanked and compensated method, using a kinetic colorimetric assay on the Roche/Hitachi Modular P analyser (Hoffman-LaRoche Inc. Ltd, Basel, Switzerland) [11]. 

All participants were classified into 1 of 3 eGFR categories: eGFR ≥ 60 ml/min/1.73 m^2^, eGFR 45 – 59 ml/min/1.73 m^2^, and eGFR < 45 ml/min/1.73 m^2^. These categories were chosen since eGFR < 45 ml/min/1.73 m^2^ is considered a more advanced state of kidney disease within Stage 3 CKD [13] and < 2% of participants fell into the category of Stage 4 CKD. Each eGFR category was then further subdivided on the basis of the intervention arm to either one of the 2 active treatment groups or the placebo group. 

### Statistical analysis

All statistical analyses were performed using SAS software version 9.2 (SAS Institute, Cary, NC, USA). Baseline clinical and biochemical characteristics of study participants were grouped by the 3-eGFR levels. Differences among eGFR levels were tested by the non-parametric Kruskal-Wallis test for continuous variables and by the χ^2^-statistics for categorical variables. 

For analysis examining the mean rate of changes in distal radius BMD over time, the placebo group and both active treatment groups were examined separately following the design of the DECALYOS II study [11]. A mixed model was performed on the repeated BMD measurements. The covariance structure (ANCOVA) for the repeated measures over time was modeled as autoregressive. Time was entered as a linear term with one degree of freedom. Two-way interactions between treatment, time, and eGFR level (i.e., time by treatment, time by eGFR, and treatment by eGFR) were entered into the model, as was the single 3-way interaction. The 3-way interaction was not significant. Only the two-way interaction between treatment and time was significant and then was retained in the final model. The mixed model was performed at each time point (12 and 24 months) to examine the effects of both eGFR and active treatments on BMD. All models were adjusted for baseline values of BMD, iPTH and bone alkaline phosphatase. 

## Results 

Of the 610 elderly women (mean age 85 y) enrolled in the DECALYOS II study, 100 (16.4%) had an eGFR < 45 ml/min/1.73 m^2^, 222 (36.4%) had an eGFR of 45 – 59 ml/min/1.73 m^2^, and 288 (47.2%) had an eGFR ≥ 60 ml/min/1.73 m^2^. At baseline, age and intact PTH level were the only two characteristics with significant differences across the 3-eGFR groups (as specified in Table 1). The remaining baseline characteristics, which included body mass index, daily calcium intake, daily vitamin D_3_ intake, serum calcium level, serum phosphorus level, serum bone-specific alkaline phosphatase level, serum 25(OH)D level, and radius BMD, were not significantly different among the 3 eGFR groups (Table 1). Furthermore, within each eGFR level no significant differences in participant’s characteristics including BMD were observed at baseline between the active treatment groups and the placebo group (supplemental Table 1). 

**Figure 1.**
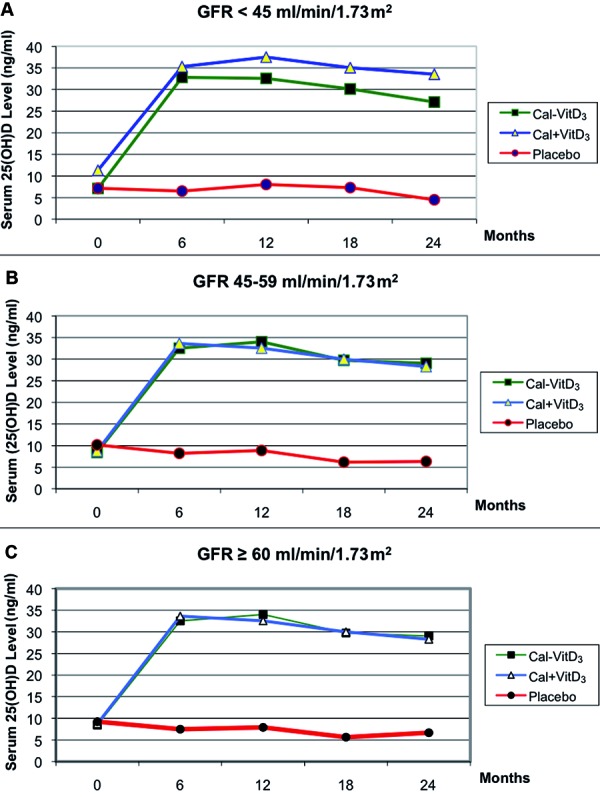
Effect of treatments on serum 25-hydroxyvitamin D levels per estimated glomerular filtration rate (eGFR) (p < 0.0001 for treatment effect). A: eGFR < 45 ml/min/1.73 m. Number of participants on each treatment arm (placebo, Ca + D, Ca-D) at baseline, 12 and 24 months were n = 100 (38, 36, 26), n = 78 (30, 27, 21) and n = 66 (28, 20, 18), respectively. B: eGFR 59 – 45 ml/min/1.73 m. Number of participants on each treatment arm (placebo, Ca + D, Ca-D) at baseline, 12 and 24 months were n = 222 (70, 73, 79), n = 182 (49, 66, 67) and n = 172 (48, 57, 67), respectively. C: eGFR ≥ 60 ml/min/1.73 m. Number of participants on each treatment arm (placebo, Ca + D, Ca-D) at baseline, 12 and 24 months were n = 288 (97, 90, 101), n = 250 (84, 79, 87) and n = 234 (76, 78, 80), respectively.

Serum 25(OH)D levels increased markedly in both active treatment groups when compared to the placebo group across all kidney function groups. The mean serum 25(OH)D levels for each eGFR group and active treatment arm are shown in Figure 1. For example, the group of participants with eGFR < 45 ml/min/1.73 m^2^, receiving either fixed combination vitamin D_3_ and calcium (Ca-D_3_) or separate combination vitamin D_3_ and calcium (Ca + D_3_), resulted in an average increase in serum 25(OH)D levels of ~ 26 ng/ml and ~ 24 ng/ml, respectively, after 6 months of oral vitamin D_3_ supplementation. Conversely, participants with an eGFR < 45 ml/min/1.73 m^2^ receiving placebo did not show any significant change in serum 25(OH)D levels. Similar results were observed among participants with eGFR 45 – 59 ml/min/1.73 m^2^ and among those with eGFR ≥ 60 ml/min/1.73 m^2^ (p < 0.0001 for all). Of note, the increase in serum 25(OH) D level was noted to occur already within 6 months of the start of vitamin D_3_ supplementation in all 3 eGFR groups. 

**Figure 2.**
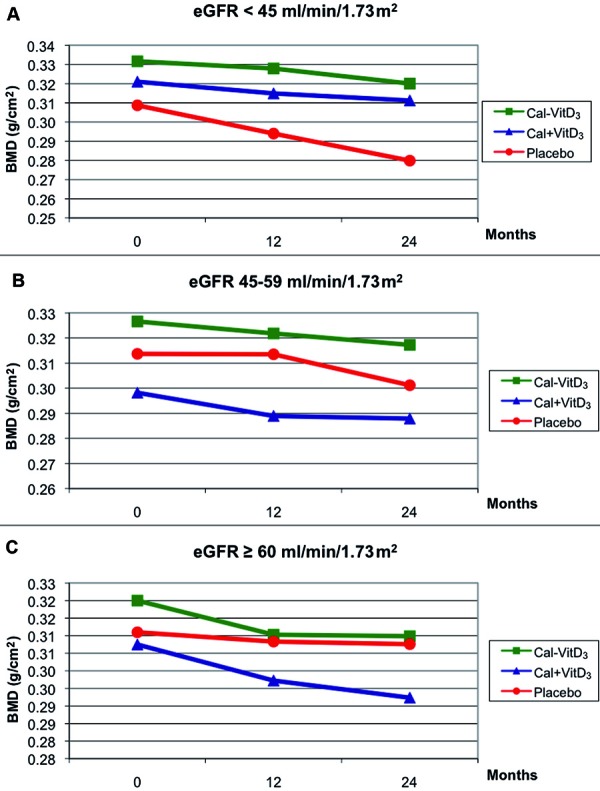
Effect of treatments on bone mineral density (BMD) of the distal radius per estimated glomerular filtration rate (eGFR) (p = 0.005 for treatment effect). A: eGFR < 45 ml/min/1.73 m. Number of participants on each treatment arm (placebo, Ca + D, Ca-D) at baseline, 12 and 24 months were n = 100 (38, 36, 26), n = 78 (30, 27, 21) and n = 66 (28, 20, 18), respectively. B: eGFR 59 – 45 ml/min/1.73 m. Number of participants on each treatment arm (placebo, Ca + D, Ca-D) at baseline, 12 and 24 months were n = 222 (70, 73, 79), n = 182 (49, 66, 67) and n = 172 (48, 57, 67), respectively. C: eGFR ≥ 60 ml/min/1.73 m. Number of participants on each treatment arm (placebo, Ca + D, Ca-D) at baseline, 12 and 24 months were n = 288 (97, 90, 101), n = 250 (84, 79, 87) and n = 234 (76, 78, 80), respectively.

The analysis of covariance for repeated measures clearly demonstrated an overall treatment effect on BMD of the distal radius (p = 0.005), with the fixed combination vitamin D_3_ and calcium (Ca-D_3_), showing higher BMD values in all eGFR groups (Figure 2). The BMD changes over time were significantly different by treatment group as suggested by the time by treatment group interaction (p = 0.02). Of note, the main effect for time showed a strong and consistent decline in BMD over time, regardless of the treatment in all eGFR groups. Finally, the effect of treatment did not appear to differ significantly by baseline kidney function group (eGFR by treatment group interaction was not significant; p = 0.25). However, the fixed combination of vitamin D_3_ and calcium (Ca-D_3_) in participants with eGFR < 45 ml/min/1.73 m^2^ showed the most favorable results (p = 0.024). 

At the end of the study (24 months), the change from baseline in serum calcium level was only slightly different in the separate combination vitamin D_3_ and calcium group (Ca + D_3_), but not in the group randomized to fixed combination vitamin D_3_ and calcium (Ca-D_3_) when compared to placebo across all kidney function groups. No significant changes were also observed in serum phosphorus levels (data not shown). 

## Discussion 

This post-hoc analysis of the DECALYOS II trial results shows that daily supplementation with 800 IU of vitamin D_3_ in combination with 1,200 mg of calcium, either as a fixed or separate combination, significantly improves serum 25(OH)D levels and reduces the rate of BMD loss of the distal radius in elderly institutionalized women with moderate CKD and severe vitamin D deficiency, independently of their baseline serum iPTH and bone-specific alkaline phosphates levels. 

Treatment effect for serum 25(OH)D levels was highly significant in all eGFR groups. In contrast, although the effectiveness of oral vitamin D_3_ supplementation on BMD of the distal radius did not vary according to level of kidney function, the estimates of the treatment effect on BMD was strongest among participants with a baseline eGFR < 45 ml/min/1.73 m^2^. A plausible explanation of this finding is that participants with more advanced kidney disease may derive more benefits by calcium/vitamin D_3_ supplementation, as this patient population is more likely to develop BMD loss over time. 

Previous observational studies have shown a significant positive relationship between serum 25(OH)D levels and BMD in the general population [14, 15]. In addition, Mucsi and colleagues reported a significant positive relationship of serum 25(OH)D levels with BMD at the radius and with broadband ultrasound attenuation at the heel (as measured with calcaneal quantitative bone ultrasound) in CKD patients requiring chronic hemodialysis [10]. However, in such study, patients with early stages of CKD were not included. Furthermore, no significant association was found between low levels of 25(OH)D and increased bone turnover as measured by serum bone markers [10]. 

In the present study, we were able to show that oral calcium with vitamin D_3_ supplementation (800 IU/d) significantly improved serum 25(OH)D levels and decreased the rate of BMD loss of the distal radius in elderly institutionalized patients with moderate CKD. These findings extend our previous observations of beneficial effects of oral vitamin D_3_ supplementation on serum iPTH across kidney function levels [16]. 

The National Kidney Foundation (NKF) Kidney Disease Outcomes Quality Initiative (K/DOQI) Clinical Practice Guidelines for Bone Mineral Metabolism and Disease [17] and Kidney Disease: Improving Global Outcomes (KDIGO) guidelines [18] recommended to maintain a serum 25(OH)D level greater than 30 ng/ml (75 nmol/l) either with ergocalciferol or cholecalciferol in CKD patients for the correction of 25(OH)D deficiency and for the initial management of secondary hyperparathyroidism. Our work shows that serum 25(OH)D levels close to that guideline can be achieved with daily oral vitamin D_3_ supplementation and can also exert beneficial effects on BMD. 

Although it is not still entirely clear how increased serum 25(OH)D levels may improve BMD in patients with CKD (i.e., a pathological condition characterized by impaired 1α-hydroxylation), there are some potential mechanisms that fit with previous experimental research. Previous studies have demonstrated that several extra-renal tissues may produce 1α-hydroxylase [19]. Thus, it is possible that bone is able to locally convert 25(OH)D to 1,25(OH)_2_D, the active metabolite of vitamin D. Alternatively, it is also possible that 25(OH)D directly stimulates bone metabolism without hydroxylation to 1,25(OH)_2_D [20]. Nonetheless, further experimental research is required to further elucidate the exact mechanisms involved. 

While our results may be of clinical relevance, they are constrained by some important limitations. First, our research is a post-hoc analysis of the data from the DECALYOS II study, and therefore, should be considered as hypothesis generating and should be further confirmed in other intervention clinical trials. Second, the study population is limited to a large group of elderly institutionalized women with moderate CKD and severe vitamin D deficiency, which it makes difficult the generalizability of our results to the broader community of patients with CKD. Third, the original study was not specifically designed to look at vitamin D_3_ supplementation by CKD stages and, therefore, participants were not randomized by kidney function level. Fourth, in these analyses the independent effects of oral calcium supplementation and vitamin D_3_ administration cannot be separated. Finally, although bone loss has been clinically associated with higher rates of fractures [21], we did not directly analyze this outcome, as fracture data were not available in our dataset. 

Future work on oral vitamin D_3_ supplementation and BMD in people with CKD should continue to address both clinical outcomes and mechanisms of action. A larger, placebo-controlled, randomized clinical trial would be helpful to further confirm our results. Such a trial could be expanded to look beyond BMD to both fracture rates and mortality. Furthermore, a better elucidation of the molecular mechanisms driving the beneficial effects of vitamin D_3_ supplementation on bone metabolism, especially in patients with CKD, is also needed to further guide therapy. 

In conclusion, our post-hoc analysis of the DECALYOS II study demonstrates that daily supplementation with 800 IU of vitamin D_3_ in combination with 1,200 mg of calcium, either as a fixed or separate combination, significantly increase serum 25(OH)D levels and radius BMD in elderly institutionalized women with moderate CKD and severe vitamin D_3_ deficiency. Collectively, these data together with previous double-masked placebo-controlled studies, support the treatment of all clinical situations associated with hypovitaminosis D_3_ and inadequate dietary calcium intake. 

**Table 1.** Demographic and baseline characteristics by estimated glomerular filtration rate (n = 610).

Characteristics	eGFR ≥ 60 ml/min/1.73 m^2^	eGFR 45 – 59 ml/min/1.73 m^2^	eGFR < 45 ml/min/1.73 m^2^	p value for trend
eGFR (ml/min/1.73 m^2^)	72 ± 10	53 ± 4	37 ± 7	
n (%)	288 (47.2)	222 (36.4)	100 (16.4)	
Age (years)	84 ± 7	86 ± 7	87 ± 7	< 0.001
Weight (kg)	58 ± 12	60 ± 12	60 ± 13	0.13
Height (cm)	155 ± 7	154 ± 7	155 ± 7	0.52
Body mass index (kg/m^2^)	25 ± 5	25 ± 5	25 ± 5	0.24
Calcium intake (mg/day)	557 ± 244	555 ± 221	594 ± 262	0.33
Vitamin D intake (IU/day)	40 ± 26	41 ± 29	43 ± 30	0.35
Serum calcium (mg/dl)	9.1 ± 0.4	9.3 ± 0.4	9.2 ± 0.4	0.80
Serum phosphorus (mg/dl)	3.2 ± 0.5	3.2 ± 0.4	3.3 ± 0.5	0.40
Bone alkaline phosphatase (ng/ml)	16 ± 16.8	15.3 ± 9.1	15.8 ± 12.5	0.22
25(OH)D (ng/ml)	8.8 ± 6.4	9.1 ± 5.8	8.7 ± 6.6	0.50
iPTH (pg/ml)	62.3 ± 44.3	72.7 ± 67.4	86.3 ± 60	< 0.001
Bone mineral density (g/cm^2^)	0.3 ± 0.1	0.3 ± 0.1	0.3 ± 0.1	0.80

All values are expressed as means ± SD. eGFR = estimated glomerular filtration rate; 25(OH)D = 25-hydroxyvitamin D; iPTH = intact parathyroid hormone.

**Supplemental Table 1.** Demographic and baseline characteristics by treatment arm within kidney function group.

Characteristics	Placebo	Ca + D_3_	Ca-D_3_	p value
eGFR ≥ 60 ml/min/1.73 m^2^				
n	97	90	101	0.48
Age (y)	84 ± 8	83 ± 7	84 ± 6	0.54
Weight (kg)	59 ± 11	58 ± 13	58 ± 12	0.67
Height (cm)	154 ± 7	155 ± 7	155 ± 7	0.78
BMI (kg/m^2^)	25 ± 5	24 ± 5	24 ± 5	0.49
Calcium intake (mg/d)	559 ± 242	561 ± 254	553 ± 237	0.98
Vitamin D intake (IU/d)	40 ± 26	41 ± 28	40 ± 25	0.97
Calcium (mg/dl)	9.2 ± 0.5	9.2 ± 0.5	9.2 ± 0.5	0.63
Phosporus (mg/dl)	3.2 ± 0.5	3.3 ± 0.5	3.2 ± 0.5	0.38
Bone alkaline phosphatase (U/l)	18 ± 8	15 ± 13	15 ± 8	0.85
25(OH)D (ng/ml)	9 ± 8	9 ± 6	9 ± 5	0.89
iPTH (pg/ml)	63 ± 50	58 ± 31	65 ± 48	0.99
Bone mineral density (g/cm^2^)	0.31 ± 0.06	0.31 ± 0.07	0.32 ± 0.07	0.54
eGFR (ml/min/1.73 m^2^)	73 ± 11	73 ± 10	70 ± 8	0.08
eGFR 45 – 59 ml/min/1.73 m^2^				
n	70	73	79	0.48
Age (y)	87 ± 7	86 ± 7	86 ± 7	0.45
Weight (kg)	61 ± 12	59 ± 13	59 ± 12	0.48
Height (cm)	155 ± 8	154 ± 8	154 ± 7	0.81
BMI k/m^2^)	25 ± 5	25 ± 5	25 ± 5	0.74
Calcium intake (mg/d)	582 ± 255	536 ± 212	547 ± 195	0.62
Vitamin D intake (IU/d)	42 ± 28	38 ± 26	43 ± 33	0.79
Calcium (mg/dl)	9.2 ± 0.4	9.2 ± 0.5	9.3 ± 0.4	0.10
Phosporus (mg/dl)	3.2 ± 0.42	3.2 ± 0.5	3.3 ± 0.4	0.72
Bone alkaline phosphatase (U/l)	16 ± 9	16 ± 11	15 ± 8	0.90
25(OH)D (ng/ml)	10 ± 7	8 ± 5	9 ± 6	0.33
iPTH (pg/ml)	73 ± 36	74 ± 47	71 ± 99	0.06
Bone mineral density (g/cm^2^)	0.31 ± 0.07	0.30 ± 0.07	0.32 ± 0.08	0.09
eGFR (ml/min/1.73 m^2^)	53 ± 4	52 ± 4	54 ± 4	0.13
eGFR < 45 ml/min/1.73 m^2^				
n	38	26	26	0.48
Age (y)	88 ± 8	87 ± 6	87 ± 6	0.86
Weight (kg)	60 ± 14	59 ± 11	61 ± 12	0.70
Height (cm)	155 ± 8	155 ± 7.4	157 ± 6	0.27
BMI (kg/m^2^)	25 ± 6	25 ± 4	25 ± 6	0.96
Calcium intake (mg/d)	552 ± 264	555 ± 245	709 ± 257	0.06
Vitamin D intake (IU/d)	40 ± 37	41 ± 27	52 ± 20	0.07
Calcium (mg/dl)	9.3 ± 0.4	9.0 ± 0.5	9.0 ± 0.5	0.09
Phosphorus (mg/dl)	3.3 ± 0.4	3.3 ± 0.4	3.2 ± 0.5	0.65
Bone alkaline phosphatase (U/l)	18 ± 17	14 ± 6	15 ± 11	0.30
25(OH)D (ng/ml)	7 ± 4	11 ± 9	7 ± 5	0.06
iPTH (pg/ml)	86 ± 53	83 ± 40	92 ± 80	0.78
Bone mineral density (g/cm^2^)	0.31 ± 01	0.32 ± 0.1	0.3 ± 0.1	0.68
eGFR (ml/min/1.73 m^2^)	38 ± 5	38 ± 5	39 ± 4	0.74

All values are expressed as mean ± SD.BMI = body mass index; 25(OH)D = 25 hydroxyvitamin D; iPTH = intact parathyroid hormone level. eGFR = estimated glomerular filtration rate.
